# Post-vaccination anti-HBs testing among healthcare workers: More economical than post-exposure management for Hepatitis B*

**DOI:** 10.1590/1518-8345.3534.3278

**Published:** 2020-06-19

**Authors:** Camila Lucas de Souza, Thaís de Arvelos Salgado, Tatiana Luciano Sardeiro, Hélio Galdino, Alexander Itria, Anaclara Ferreira Veiga Tipple

**Affiliations:** 1 Secretaria Municipal de Saúde de Goiânia, Escola Municipal de Saúde Pública de Goiânia, Goiânia, GO, Brazil.; 2 Universidade Federal de Goiás, Hospital das Clínicas, Goiânia, GO, Brazil.; 3 Secretaria Municipal de Saúde de Goiânia, Centro de Referência em Saúde do Trabalhador de Goiânia, Goiânia, GO, Brazil.; 4 Universidade Federal de Goiás, Faculdade de Enfermagem, Goiânia, GO, Brazil.; 5 Universidade Federal de Goiás, Instituto de Patologia Tropical em Saúde Pública, Goiânia, GO, Brazil.

**Keywords:** Occupational Exposure, Health Personnel, Hepatitis B Vaccines, Hepatitis B Antibodies, Costs and Cost Analysis, Health Care Costs, Exposição Ocupacional, Pessoal de Saúde, Vacinas contra Hepatite B, Anticorpos Anti-Hepatite B, Custos e Análise de Custo, Custos de Cuidados de Saúde, Exposición Ocupacional, Personal de Salud, Vacunas contra Hepatitis B, Anticuerpos contra la Hepatitis B, Costos y Análisis de Costo, Costos de la Atención en Salud

## Abstract

**Objective:**

to compare the direct cost, from the perspective of the Unified Health System, of assessing the post-vaccination serological *status* with post-exposure management for hepatitis B among health care workers exposed to biological material.

**Method:**

cross-sectional study and cost-related, based on accident data recorded in the System of Information on Disease Notification between 2006 and 2016, where three post-exposure and one pre-exposure management scenarios were evaluated: A) accidents among vaccinated workers with positive and negative serological *status* tests for hepatitis B, exposed to known and unknown source-person; B) handling unvaccinated workers exposed to a known and unknown source-person; C) managing vaccinated workers and unknown serological *status* for hepatitis B and D) cost of the pre-exposure post-vaccination test. Accidents were assessed and the direct cost was calculated using the decision tree model.

**Results:**

scenarios where workers did not have protective titles after vaccination or were unaware of the serological *status* and were exposed to a positive or unknown source-person for hepatitis B.

**Conclusion:**

the direct cost of hepatitis B prophylaxis, including confirmation of serological *status* after vaccination would be more economical for the health system.

## Abstract (pt)

**Objetivo:**

comparar o custo direto, sob a perspectiva do Sistema Único de Saúde, da avaliação do *status* sorológico pós-vacinação com o manejo pós-exposição para hepatite B entre trabalhadores da área da saúde expostos ao material biológico.

**Método:**

estudo transversal e de custo, realizado a partir dos dados de acidentes registrados no Sistema de Informação de Agravos de Notificação entre 2006 e 2016, em que foram avaliados três cenários de manejo pós-exposição e um de pré-exposição: A) acidentes entre trabalhadores vacinados com *status* sorológico positivo e negativo para hepatite B, expostos à pessoa-fonte conhecida e desconhecida; B) manejo dos trabalhadores não vacinados expostos à pessoa-fonte conhecida e desconhecida; C) manejo dos trabalhadores vacinados e *status* sorológico desconhecido para hepatite B e D) custo do teste pós vacinação pré-exposição. Os acidentes foram avaliados e o custo direto foi calculado utilizando o modelo árvore de decisão.

**Resultados:**

apresentaram maior custo os cenários em que os trabalhadores não possuíam títulos protetores após a vacinação ou desconheciam o *status* sorológico e foram expostos à pessoa-fonte positivo ou desconhecida para hepatite B.

**Conclusão:**

o custo direto da profilaxia para hepatite B, incluindo a confirmação do *status* sorológico após vacinação seria mais econômico para o sistema de saúde.

## Abstract (es)

**Objetivo:**

comparar el costo directo, desde la perspectiva del Sistema Único de Salud, de la evaluación del *status* serológico post-vacunación con el manejo post-exposición para la hepatitis B entre los trabajadores de la salud expuestos a material biológico.

**Método:**

estudio transversal y de costos, basado en datos de accidentes registrados en el Sistema de Información de Enfermedades Notificables entre 2006 y 2016, en el que se evaluaron tres escenarios de gestión posteriores a la exposición y uno previo a la exposición: A) accidentes entre trabajadores vacunados con *status* serológico positivo y negativo para hepatitis B, expuestos a una fuente de origen conocida y desconocida; B) manejo de trabajadores no vacunados expuestos a una fuente conocida y desconocida; C) manejo de trabajadores vacunados y estado serológico desconocido para hepatitis B y D) costo de la prueba de pre-exposición post-vacunación. Se evaluaron los accidentes y se calculó el costo directo utilizando el modelo de árbol de decisión.

**Resultados:**

los escenarios en los que los trabajadores no tenían títulos de protección después de la vacunación o desconocían el *status* serológico y estaban expuestos a una persona fuente positiva o desconocida para la hepatitis B reflejaron un costo más alto.

**Conclusión:**

el costo directo de la profilaxis para la hepatitis B, incluida la confirmación del *status* serológico después de la vacunación sería más económico para el sistema de salud.

## Introduction

In the world, approximately 257 million individuals live with chronic hepatitis B virus (HBV) infection^(^ 1 ^)^. It is known that the cost of treating this disease is high^(^ 2 ^-^ 3 ^)^.

In Brazil, 233,027 confirmed hepatitis B cases were reported in the period from 1999 to 2018, with detection rates of 6.7/100,000 inhabitants in 2018, in which 0.3% of the transmission occurred through the occupational route^(^ 4 ^)^.

Infection by occupational exposure can occur during accidents with biological material among Health Care Workers (HCW), according to studies that show rates of 17.3% to 58.4% in Brazil^(^ 5 ^-^ 6 ^)^ and 36,7 % to 78,0% in other countries^(^ 7 ^-^ 9 ^)^.

In view of the risk of exposure to HBV, the main preventive measure is the vaccination^(^ 10 ^)^. In Brazil, the Unified Health System (SUS - abbreviation in Portuguese) bears the costs of the HBV vaccine within the National Immunization Program, making it available free of charge since 1998^(^ 11 ^)^.

The vaccine is safe and effective, ensuring 92% protection for immunocompetent adults^(^ 12 ^)^. Despite the high protection, it is recommended after vaccination to perform antibodies against surface antigen (anti-HBs) to confirm immunity to the virus^(^ 10 ^)^.

Unlike the HBV vaccine, the anti-HBs test is not routinely available in the public health system after vaccination in Brazil.

In handling the accident with biological material, considering the recommendations of the *Centers for Disease Control and Prevention* (CDC)^(^ 10 ^)^ and the Ministry of Health in Brazil^(^ 13 ^)^, evaluation of vaccination history and serological *status* is required for the hepatitis B of HCW and HBV serological *status* testing for hepatitis B by the surface antigen (HBsAg) from the known source-person at the time of occupational exposure.

After this assessment at the time of the accident, four approaches can be adopted considering the *Guidance for Evaluating Health-Care Personnel for Hepatitis B Virus Protection and for Administering Postexposure Management protocol* - CDC^(^ 10 ^)^. Depending on the serological *status* of the source-person and the victim, the conducts are: No conduct, realizing the vaccine, realizing the vaccine and administering a dose of Hyperimmune Immunoglobulin for Hepatitis B (IGHAHB) and administering two doses of IGHAHB. In the last three aforementioned conducts, the injured worker must perform the anti-HBs test after the vaccine one to two months after the last dose and after four to six months of this immunoglobulin^(^ 10 ^)^.

The management of accidents with biological material among HCW is expensive in several countries, mainly in percutaneous exposures^(^ 14 ^-^ 18 ^)^. Although the performance of the anti-HBs test among these workers is a recommendation of the Ministry of Health and Labor through the Regulatory Norm (NR) 32/2005^(^ 19 ^-^ 20 ^)^ and the CDC^(^ 10 ^)^, it is known that a considerable part of the vaccinated HCW ignore the serological *status* for the HBV^(^ 5 ^,^ 21 ^-^ 23 ^)^. Ignoring this *status* at the time of the accident with a positive source-person requires a high-cost intervention with immunoglobulin, which would turn expensive the post-exposure handling related to the HBV^(^ 15 ^)^.

In the economic studies, the direct cost involves technology costs for health interventions, including drugs and exams^(^ 24 ^-^ 25 ^)^. The evaluation of costs in the health area is increasingly present in the management of health services; therefore, good quality scientific evidence on costs and health outcomes helps in the decision-making^(^ 26 ^)^.

Since the post-vaccination anti-HBs test is not routinely offered to the worker free of charge by SUS, it was asked what is the lowest cost related to occupational exposure to HBV?

In this sense, the aim of this study was to compare the direct cost, from the perspective of the Unified Health System, of assessing the post-vaccination serological status with post-exposure management for hepatitis B among health care workers exposed to biological material.

## Method

Cross-sectional, descriptive and partial economic evaluation study, focusing on the direct cost of occupational post-exposure management to biological material. The study’s population was HCW that suffered accident with exposure to biologic material notified in the database of Aggravated Notification Information System (SINAN-NET), in the municipality of Goiânia, in the period from 2006 to 2016, which corresponds to the beginning of the notifications from the municipality until the last year in which the data were completed and marked a 10-year period of notifications.

The study site is located in the Midwest region of Brazil. According to the Brazilian Institute of Geography and Statistics in 2017, this municipality had 1,466,105 inhabitants^(^ 27 ^)^. There were 3,281 health facilities (public, philanthropic and private networks) and 25,367 HCW working in the health services^(^ 28 ^)^.

To evaluate the direct cost of performing the anti-HBs test and the management after exposure to the HBV, the analyzed epidemiological variables were: gender, age, education, professional category, biological material involved, object involved, type of exposure, hepatitis B vaccine of HCW, HCW anti-HBs test, identification of the source-person and HBsAg of the source-person.

From such data, four scenarios were evaluated, representing the possibilities of intervention considering the serological *status* of the source-person and injured HCW (A, B, C, D), considering the recommendations of CDC^(^ 10 ^)^, adopted as a reference for providing greater protection to the workers. In scenario A and B, the direct costs for HBV-related post-exposure management were quantified among HCW exposed to biological material from real data, considering the previous vaccination and the result of the worker’s anti-HBs test performed at the time of accident (scenario A) or the non-vaccination of the same (scenario B). While, in scenario C, the costs of post-exposure management for HBV by simulation were measured, considering epidemiological studies. In this scenario, the HCW did not know the result of the anti-HBs test at the time of occupational exposure, and for the simulation one considered the immunogenicity rate of 92%^(^ 12 ^)^, the known source-person rate of 73%^(^ 21 ^,^ 29 ^-^ 31 ^)^ and the prevalence of positive HBsAg source-person of 1.0%^(^ 21 ^,^ 30 ^-^ 31 ^)^.

In scenario D, the direct costs of the HBV prevention measure were measured by performing the anti-HBs test 30 days after the last dose of the vaccine, considering that the injured HCW had performed this primary post-vaccination test and before the accident with biological material, considering the same immunogenicity rate as scenario C.

This study evaluated the direct cost from SUS perspective, according to the current table of values of the Management System of the Table of Procedures, Medicines and Orthotics, Prostheses and Special Materials (SIGTAP).

For the calculations, we first used the Brazilian currency in Reais (R$) that was converted to the U.S. dollar (US$) with a value of 1 US$=R$ 3.26, based on the price of 07/15/2016, available on the *site* of the Central Bank of Brazil.

The values of the technologies (unitary costs) used in this study were US$ 5.69 the anti-HBs test, US$ 5.69 the HbsAg test, US$ 259.75 the IGHAHB of 500 International Units (IU) and US$ 3.07 medical consultation in worker’s health. The costs were calculated considering the number of HCW multiplied by the value of the test or technology (anti-HBs, HBsAg, IGHAHB and medical consultation) in each scenario.

The cost of the vaccine was not considered in this study for economic analysis, as it was assumed that it would not bring financial impact, since this cost is predicted by the SUS for all HCW^(^ 11 ^)^.

Epidemiological data were processed and analyzed by Statistical Package for the Social Sciences (SPSS^®^), version 20.0 using descriptive statistics. The following criteria were considered for data analysis based on the CDC recommendation^(^ 10 ^)^:

- Vaccinated HCW - those who received the three doses of hepatitis B vaccine reported by the worker;
- Non-vaccinated HCW - those who did not receive the three doses of the vaccine, vaccine situation ignored and without information;
- HCW with protective titers to HBV - those with anti-HBs test >10 IU/ millilitres (ml);
- HCW without protective titers to HBV - those with anti-HBs test;
- Unknown source-person - those with inconclusive HBsAg test, not performed, ignored and without information, whose management, recommended by the CDC^(^ 10 ^)^, is the same for those with positive HBsAg;
- Vaccinated HCW and with unknown anti-HBs test - those with inconclusive test, not performed, ignored or without information.
- For the cost analysis of IGHAHB, the prescription of 500 IU was considered as the standard dose, since it is the minimum dosage prescribed for adults^(^ 32 ^)^.

The economic analysis used was the decision tree model; this graphic representation begins from the left with a decision node, which is divided into branches that propose to evaluate comparatively. In each branch, the probabilities of events must be described until the final event. Therefore, a series of probability nodes appear in each branch. At the end of these branches, the outcomes are presented as terminal node, indicating the final impacts of each branch with their respective costs associated with each analyzed event^(^ 33 ^-^ 34 ^)^.

The approach used for the analysis was macrocosting or some *top-down* method, which allows for a cost analysis of secondary data retrospectively^(^ 24 ^)^.

A study approved in the research ethics committee of Clinical Hospital, Federal University of Goiás, under protocol no. 41425/2013.

## Results

There were recorded 7,265 accidents with biological material among HCW in the city of Goiânia from 2006 to 2016, aged from 21 - 30 years old (39.3%), with a predominance of females (80.5%) and with high school education (43.0%). The most exposed team was nursing (55.2%), followed by the physician (10.2%).

Regarding the profile of accidents with biological material, percutaneous exposures predominated (72.4%) in the presence of blood (74.4%), and the most involved objects were needles with and without lumen (62.1%).

For post-exposure management of biological material it is necessary to know the vaccination history against hepatitis B and serological *status* (anti-HBs test) of the HCW at the time of the accident and the serological *status* (HBsAg) of the source-person when known. Regarding the vaccination history against hepatitis B, it was recorded in the accident notification form that 6,184 (85.1%) workers had received all three doses of the vaccine, 542 (7.5%) were not vaccinated or did not complete the vaccination schedule and in 539 (7.4%) cases there was no record of this information.

**Figure 1:**
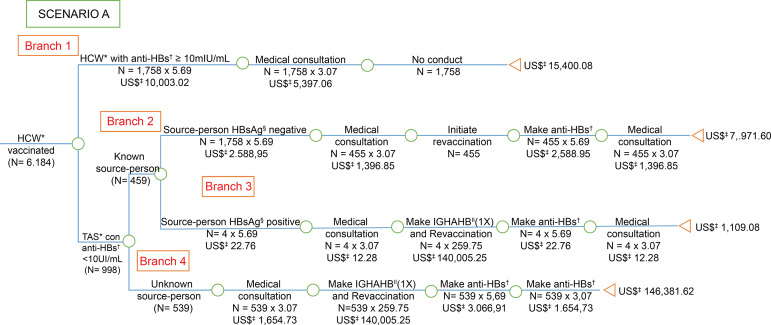
Economic analysis of post-exposure management for hepatitis B among health care workers, victims of accidents with biological material, vaccinated against hepatitis B (3 doses) and anti-HBs at the time of the accident, considering the recommendations of the *Centers for Disease Control and Prevention*. Goiânia, GO, Brazil, 2006-2016 ^*^HCW = Health Care Worker; ^†^anti-HBs = Antibody against hepatitis B virus surface antigen; ^‡^US$ = Conversion rate: 1 US$=3,26 in 07/15/2016; ^§^HBsAg = Surface antigen for hepatitis B; ^||^IGHAHB = Immunoglobulin hyper-immune for hepatitis B

Regarding serological *status* to HBV, of the 6,184 vaccinated HCW (Figure 1), 2,756 (44.6%) performed the anti-HBs test, of which 1,758 (63.8%) had protective titers at the time of the accident and 998 (36.2%) didn’t have it. Of the 3,428 workers who did not undergo the anti-HBs test (Figure 3), when considering the 92% immunogenicity rate for hepatitis B vaccine, it is assumed that 3,154 (92%) had protective titles for the virus and 274 (8%) would not possess.

**Figure 2:**
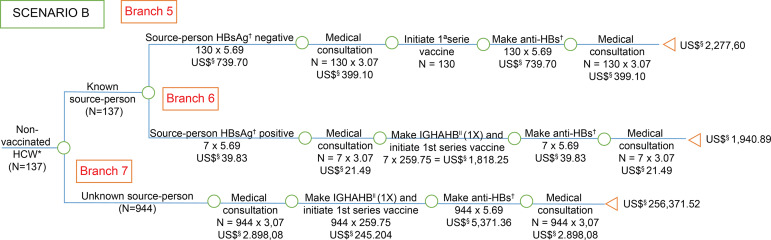
Economic analysis of post-exposure management for hepatitis B among health care workers, victims of accidents with biological material, not vaccinated against hepatitis B (3 doses), exposed to known and unknown source people, exposed to known and unknown source people, considering the recommendations of the *Centers for Disease Control and Prevention*. Goiânia, GO, Brazil, 2006-2016 ^*^HCW = Health Care Worker; ^†^HBsAg = Surface antigen for hepatitis B ; ^‡^anti-HBs = Antibody against hepatitis B virus surface antigen; ^§^US$ = Conversion rate: 1 US$=3.26 in 07/15/2016; ^||^IGHAHB = Immunoglobulin hyper-immune for hepatitis B

**Figure 3:**
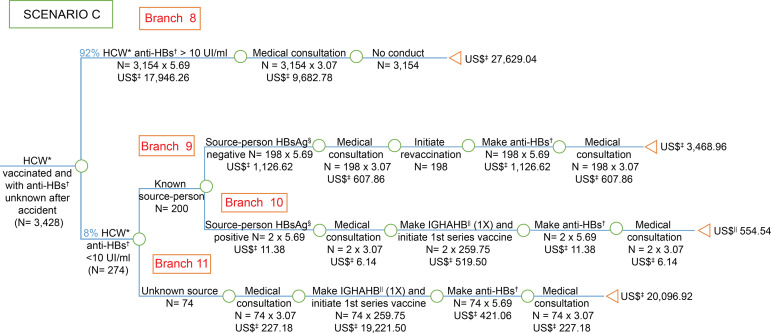
Economic analysis of the simulation of occupational post-exposure management of biological material for hepatitis B among health care workers, victims of accidents with biological material, vaccinated and with unknown anti-HBs test after the accident with biological material, considering the recommendations of the *Centers for Disease Control and Prevention*. Goiânia, GO, Brazil, 2006-2016 ^*^HCW = Health Care Worker; ^†^anti-HBs = Antibody against hepatitis B virus surface antigen; ^‡^US$ = Conversion rate: 1 US$=3,26 in 07/15/2016; ^§^HBsAg = Surface antigen for hepatitis B; ^||^IGHAHB = Immunoglobulin hyper-immune for hepatitis B

Regarding the serological *status* (HBsAg) of the source-person, a prevalence of positive HBsAg was observed among known source people of 1.8% (95% CI: 1.0 - 3.2). Among vaccinated HCW against hepatitis B and with anti-HBs <10 IU\/ml, the prevalence of positive HBsAg with known source-person was 0.9% (95% CI: 0.3 - 2.0) and among unvaccinated HCW, the prevalence of positive HBsAg with known source-person was 5.1% (95% CI: 2.3 - 9.8).

**Figure 4:**
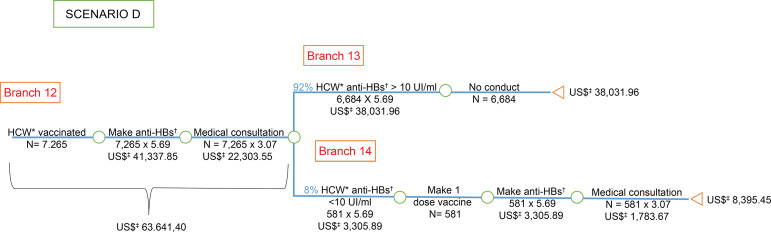
Economic analysis of the evaluation of serological *status* after primary vaccination for hepatitis B among health care workers, victims of accidents with biological material, considering the recommendations of the *Centers for Disease Control and Prevention*. Goiânia, GO, Brazil, 2006-2016 ^*^HCW = Health Care Worker; ^†^anti-HBs = Antibody against hepatitis B virus surface antigen; ^‡^US$ = Conversion rate: 1 US$=3,26 in 07/15/2016

The costs were presented in the branches of the “decision tree” model, with the costs of post-exposure management described in scenarios A (Figure 1), B (Figure 2) and C (Figure 3) and the cost of post-vaccination prevention primary in scenario D (Figure 4).

The cost of prevention measures before the accident with biological material and the management after exposure to HBV among the injured HCW in relation to vaccination history and serological *status* for the HBV of the worker and the source-person is described in Table 1.

**Table 1:** Economic analysis of prevention measures and post-exposure management among health care workers, victims of accidents with biological material in the municipality of Goiânia. Goiânia, GO, Brazil, 2006-2016

Handling situation post-exposure (n)	Dollar Costs (US$*)	
	Total	*Per capita*
**Vaccinated**		
HCW^†^ with anti-HBs^‡^ positive (1,758)	15,400.08	8.76
HCW^†^ with anti-HBs^‡^ negative with source-person HBsAg^§^ negative (455)	7,971.60	17.52
HCW^†^ with anti-HBs^‡^ negative with source-person HBsAg^§^ positive (4)	1,109.08	277.27
HCW^†^ with anti-HBs^‡^ negative with unknown source-person (539)	146,381.62	271.58
**Non-vaccinated**		
HCW^†^ with source-person HBsAg^§^ negative (130)	2,277.60	17.52
HCW^†^ with source-person HBsAg^§^ positive (7)	1,940.89	272.13
HCW^†^ with unknown source-person (944)	256,371.52	271.58
**HCW^†^ vaccinated with anti-HBs test^‡^ unknown after the accident**		
HCW^†^ with anti-HBs^‡^ positive (3.154)	27,629.04	8.76
HCW^†^ with anti-HBs^‡^ negative with source-person HBsAg^§^ negative (198)	5,203.44	26.28
HCW^†^ with anti-HBs^‡^ negative with source-person HBsAg^§^ positive (2)	554.54	277.27
HCW^†^ with anti-HBs^‡^ negative with unknown source-person (74)	20,096.92	271.58
Vaccinated with anti-HBs^‡^ primary post-vaccination (7,265)	63,641.40	8.76

* US$ = Conversion rate: 1 US$ = 3.26 in 07/15/2016;

† HCW = Health Care Worker;

‡ anti-HBs = Antibody against hepatitis B virus surface antigen;

§ HBsAg = Surface antigen for hepatitis B

## Discussion

The predominance, in this study, of accidents with biological material among female HCW, agrees with other Brazilian studies^(^ 5 ^-^ 6 ^,^ 21 ^,^ 35 ^-^ 36 ^)^ and of other countries^(^ 9 ^,^ 37 ^-^ 38 ^)^. Regarding age group, there was verified, in accordance with other researches^(^ 39 ^-^ 40 ^)^, higher prevalence of young adult workers.

As for the health team, the nursing team corresponds to the largest number of professionals in the health services, being the one who first assists the patient and is present from the admission to the discharge^(^ 41 ^)^, is responsible for numerous procedures^(^ 41 ^)^, which is why the higher incidence of accidents is inferred.

As identified in other researches^(^ 21 ^,^ 29 ^,^ 37 ^,^ 42 ^-^ 43 ^)^, in this study, it was identified that the most frequent object in accidents was the needle with and without lumen, therefore exposure to sharp objects prevailed, followed by exposure to mucous membranes. Exposures involving blood were the most numerous, as found in studies in the state of Goiás^(^ 36 ^,^ 44 ^)^, in other Brazilian states^(^ 6 ^,^ 21 ^,^ 42 ^)^ and in other countries^(^ 7 ^,^ 29 ^,^ 37 ^)^. Together, the data characterizes a population that should be the target of accident prevention campaigns and the need for investments in professional training to reduce biological occupational risk.

An essential preventive measure against HBV infection is vaccination, and the HCW needs to have it documented^(^ 10 ^,^ 13 ^)^. Studies show vaccination frequencies against hepatitis B (three doses) of 73,5% to 97,5% among HCW, victims of biological material accident^(^ 21 ^,^ 29 ^,^ 35 ^)^, the vaccination rate of this study is in this range (85.1%), showing that policies to encourage and monitor the immunization of workers are, still, fundamental and deserve the attention of the managers.

Following vaccination against hepatitis B, the performance of the anti-HBs test is essential for the safety of workers, as it will demonstrate the immunological *status* for the HBV^(^ 10 ^,^ 13 ^)^. In Brazil, the rate of carrying out this test after primary vaccination in this group varied between 30.4%^(^ 45 ^)^; 27.9%^(^ 23 ^)^ and 4.1%^(^ 36 ^)^.

The anti-HBs test is not available in all hospitals for emergencies, as in the case of accidents with biological material^(^ 46 ^)^. At the reference units for this type of care, at the study site, blood samples are collected from the injured HCW for the performance of various serologies, including anti-HBs, and the results are delivered after 30 days. Despite the recommendation to perform this test in post-exposure management^(^ 10 ^,^ 13 ^)^, its performance in this study was low (44.6%), as shown in other studies in Brazil, in which the rate of anti-HBs testing among HCW exposed to biological material, in the accident time, ranged from 14.6% to 52.8%^(^ 21 ^-^ 23 ^,^ 30 ^-^ 31 ^,^ 47 ^)^.

Regarding the serologic *status* of the source-person for HBsAg positive, a rate of 1.8% (95% CI 1.0 - 3.2) was observed in this study. Rate of 0.5% to 1.4%^(^ 21 ^,^ 30 ^-^ 31 ^)^ were identified in the literature.

In Scenario A (Figure 1), it was observed that four HCW with anti-HBs <10 IU/ml were exposed to HBsAg positive source-person; therefore the conduct recommended by the CDC^(^ 10 ^)^ is the administration of one dose of IGHAHB and one dose of the vaccine, simultaneously and as soon as possible, as the efficacy of IGHAHB, when administered after seven days of exposure, is unknown^(^ 10 ^)^. In this case, the direct cost of this group was US $ 1,109.08, corresponding to US $ 277.27 *per* worker. The direct cost could have been avoided with the second vaccination schedule followed by the anti-HBs test, as probably the number of HCW with anti-HBs <10 IU/ mL would be lower, since the worker can respond to a second schedule^(^ 10 ^)^.

The health protection of the HBP-related HCW is very explicit in Collegiate Board Resolution No. 11, which provides for the requirements of good operating practices for dialysis services, as it prohibits workers without protective titles to HBV, to carry out assistance during the session of hemodialysis and, in the processing of dialyzers and arterial and venous lines of patients with positive serology for hepatitis B^(^ 48 ^)^. However, this regulation does not apply to other areas of care, which also offer risk of contact with blood from HBV-positive patients; then it is considered necessary to encourage the performance of anti-HBs testing among all HCW.

Considering the prevention for HBV, it is interesting to note that NR 32/2005 ensures that all HCW should be provided with the hepatitis B vaccine, free of charge, with the employer must keep supporting document and keep it available for labor inspection. However, when it comes to anti-HBs testing, the standard is not so clear. Declares that the employer must monitor the effectiveness whenever recommended by the Ministry of Health and, when necessary, provide the vaccine booster^(^ 20 ^)^.

Although the vaccine is provided free by SUS, in this study, it was observed that there are still HCW without vaccination, according to scenario B (Figure 2). Therefore, it would be important for managers to provide effective strategies to ensure vaccine completion for workers prior to admission to the health service^(^ 49 ^)^.

In scenario B (Figure 2), HCW not vaccinated against hepatitis B were analyzed. These workers who had an accident with a positive HBsAg source-person had a high cost to the health system, as well as those who had an unknown source-person accident. However, scenario D (Figure 4) in which the test was performed before exposure was the one with the lowest cost when compared to the other scenarios.

Consequently, when comparing the *per capita* cost of scenario A (Figure 1) in which the HCW vaccinated with the anti-HBs test <10 IU/mL was exposed to HBsAg-positive source-person with the scenario D (Figure 4) of the vaccinated worker and with the anti-HBs test >10 IU/mL after hepatitis B vaccine before the accident with biological material, it was noted that the first cost was about 32 times more expensive for SUS (Table 1). Thus, the opportunity to allocate resources to other programs, including those aimed at the health of the own workers, is lost^(^ 20 ^)^.

When comparing the *per capita* cost of the HCW vaccinated with the anti-HBs test <10 IU/ml (scenario A - Figure 1) who had an accident with an unknown source-person with a worker vaccinated with anti-HBs after primary vaccination (scenario D - Figure 4), the cost was approximately 31 times higher. As well, when checking the *per capita* cost of the HCW vaccinated with the anti-HBs test <10 IU \/ ml (scenario A - Figure 1) exposed to HBsAg-negative source-person with the worker vaccinated with anti-HBs primary vaccination (scenario D - Figure 4), the cost was in about twice as expensive for SUS.

In cases of *post*-exposure management of unvaccinated HCW (scenario B - Figure 2), the *per capita* costs were elevated when compared also to the worker vaccinated with anti-HBs after primary vaccination (scenario D - Figure 4), being 32 times more expensive for SUS when the SAD was exposed to HBsAg-positive source-person and 31 times higher when the person-source was unknown.

When comparing the *per capita* cost of vaccinated HCW and with anti-HBs <10 IU/ml exposed to HBsAg-positive source-person (scenario C - Figure 3) with the cost of the worker vaccinated with anti-HBs after primary vaccination (scenario D - Figure 4), the cost was around 32 times higher for SUS. Still in the scenario C (Figure 3), when comparing the *per capita* cost of HCW with negative anti-HBs exposed to unknown source-person with the worker’s cost vaccinated with anti-HBs after primary vaccination (scenario D - Figure 4), the cost was 31 times more costly for SUS.

A Brazilian study showed that HBV infection has high costs for the health system, with an average annual cost per patient of U $ 117 to 11,488 depending on medication^(^ 2 ^)^, without mentioning the costs of carrying out tests for the clinical and laboratory monitoring of the injured worker. The cost for treating hepatitis B is also high in other countries^(^ 50 ^-^ 51 ^)^.

Therefore, when the health system pays for preventable disease treatments, the opportunity to invest in effective prevention and promotion measures is lost^(^ 52 ^)^. Such analysis can be performed through the opportunity cost, which represents the cost of losing the opportunity to apply financial resources in other health technologies or programs that have a positive impact on public health^(^ 53 ^)^.

This study showed that the allocation of SUS resources to preventive measures, including the provision and monitoring of anti-HBs tests to all HCW, is more economical than post-exposure management and these data can support public policies on worker health, ensuring greater security at a lower cost. Some gaps found in the SINAN-NET database were its limitation.

## Conclusion

The direct cost of post-exposure prophylaxis for SUS was about 30 times more expensive than the costs of post-vaccination testing in those accidents in which the source-person was positive or unknown and the professional had unknown anti-HBs.

Scenarios A (branch three and four), scenario B (branch six and seven) and scenario C (branch 10 and 11) for post-exposure management to HBV when compared to scenario D, which represents the primary vaccination followed by confirmation of immunity confirmed by the anti-HBs test, showed greater *per capita* cost impact.

Health managers can rely on the findings of this study for the implementation of the routine of carrying out the post-vaccination anti-HBs test, ensuring greater protection to the health of the worker with a reduction in the costs of post-exposure management related to HBV, optimizing scarce public resources in our country.
